# Supplementary material for: Syncope and subsequent traffic crash: A responsibility analysis
Source: PLoS One. 2023 Jan 19;18(1):e0279710. doi: 10.1371/journal.pone.0279710 (PMC9851499; doi:10.1371/journal.pone.0279710)
Supplement: S1 File — Table using hypothetical data to illustrate that responsibility analyses inherently account for road exposure because all crash-involved drivers were driving at the time of the crash. Grey cell highlights the change in inputs as the reader moves from left to right. Odds ratio = (C/D)÷(E/F). Scenario 1 depicts a responsibility analysis of all crashes in a population of 100,000 individuals, 1% of whom have a disease (’exposed’), with identical road exposure between exposed and control individuals. Scenario 2 depicts a responsibility analysis of all crashes among exposed and controls, but with a yearly travel distance among exposed that is half that of controls. The odds ratios produced in Scenarios 1 and 2 are identical and reflect only the relative proportion of crashes for which each group is responsible; they are unaffected by changes in cohort size and road exposure provided the probability of responsibility is not influenced by road exposure. (DOCX) [file pone.0279710.s001.docx]

**Item S1. Responsibility analysis accounts for changes in road exposure**

| **Variable** | **Scenario 1:**  **Responsibility analysis of individuals with disease**  **(~1% of population)**  **Road exposure identical among exposed and controls** | **Scenario 2:**  **Responsibility analysis of individuals with disease**  **(~1% of population)**  **Road exposure half as much among exposed relative to controls** |
| --- | --- | --- |
| **INPUT VALUES** |  |  |
| **Number of individuals** |  |  |
| Exposed | 1,000 | 1,000 |
| Control | 99,000 | 99,000 |
| **Crash risk**  **(per 100,000 km traveled)** |  |  |
| Exposed | 0.20 | 0.20 |
| Control | 0.10 | 0.10 |
| **Travel distance per year**  **(units of 100,000 km)** |  |  |
| Exposed | 1 | **0.50** |
| Control | 1 | 1 |
| **Proportion of crashes for which drivers are responsible** |  |  |
| Exposed | 0.75 | 0.75 |
| Control | 0.50 | 0.50 |
| **COMPUTED VALUES** |  |  |
| **Number of responsible crashes** |  |  |
| Exposed (C) | 150 | 75 |
| Control (D) | 4,950 | 4,950 |
| **Number of non-responsible crashes** |  |  |
| Exposed (E) | 50 | 25 |
| Control (F) | 4,950 | 4,950 |
| **Odds ratio** | 3 | 3 |
