# Supplementary material for: Syncope and subsequent traffic crash: A responsibility analysis
Source: PLoS One. 2023 Jan 19;18(1):e0279710. doi: 10.1371/journal.pone.0279710 (PMC9851499; doi:10.1371/journal.pone.0279710)
Supplement: S2 File — (DOCX) [file pone.0279710.s002.docx]

**Item S2. Data sources**

- **Laboratory data**: VCH Patient Care Information System [creator] (2018): Serum concentrations of hemoglobin, hematocrit, and troponin I. Vancouver Coastal Health [publisher]. Data Extract. VCH (2018).
- **Electrocardiogram (ECG) data**: VCH Regional MUSE™ Cardiology Information System v9 (General Electric, Boston, Massachusetts, USA) [creator] (2018): Numerical ECG data and physician ECG interpretation. Vancouver Coastal Health [publisher]. Data Extract. VCH (2018). Included all ECGs from the index ED visit and from baseline ECGs, defined as the two most recent ECGs in the VCH Regional MUSE™ system.
- **Consolidation File**: British Columbia Ministry of Health [creator] (2019): Consolidation File (MSP Registration & Premium Billing). V2. Population Data BC [publisher]. Data Extract. MOH (2018).
- **Medical Services Plan**: British Columbia Ministry of Health [creator] (2018): Medical Services Plan (MSP) Payment Information File. Population Data BC [publisher]. Data Extract. MOH (2018).
- **National Ambulatory Care Reporting System**: Canadian Institute for Health Information [creator] (2018): National Ambulatory Care Reporting System (NACRS). V2. Population Data BC [publisher]. Data Extract. MOH (2018).
- **Discharge Abstract Database**: Canadian Institute for Health Information [creator] (2019): Discharge Abstract Database (Hospital Separations). Population Data BC [publisher]. Data Extract. MOH (2018).
- **PharmaNet**: British Columbia Ministry of Health [creator] (2019): PharmaNet. V2. Population Data BC [publisher]. Data Extract. Data Stewardship Committee (2018).
- **Income Band**: Statistics Canada [creator]: Statistics Canada Income Band Data. Catalogue Number: 13C0016. V2. Population Data BC [publisher]. Data Extract. Population Data BC (2018).
- **Driver data** (Driver license, BC Traffic Accident System, ICBC Claims File): Insurance Corporation of British Columbia [creator] (2019): Driver Experience, Contraventions, and Exam tables and the Traffic Accident System. Insurance Corporation of British Columbia [publisher]. Data Extract. ICBC (2018).

We linked health and driving data using a previously established probabilistic linkage between Personal Health Number and Driver License Number based on name, sex and birthdate, with linkage rates exceeding 95% (Brubacher JR, Chan H, Erdelyi S, Zed PJ, Staples JA, Etminan M. Medications and risk of motor vehicle collision responsibility in British Columbia, Canada: a population-based case-control study. Lancet Public Health. 2021 Jun;6(6):e374-e385.).

Because of the design of the original cohort study, Data Stewards only permitted release of administrative health data from 5 years prior to and 1 year following the first syncope ED visit.

All inferences, opinions and conclusions drawn in this manuscript are those of the authors and do not reflect the opinions or policies of the Data Stewards.
