# Supplementary material for: Syncope and subsequent traffic crash: A responsibility analysis
Source: PLoS One. 2023 Jan 19;18(1):e0279710. doi: 10.1371/journal.pone.0279710 (PMC9851499; doi:10.1371/journal.pone.0279710)
Supplement: S3 File — We considered comorbidities present if identified in diagnostic coding from ≥1 hospitalization or ≥2 physician visits in a 1-year lookback interval. ICD = The World Health Organization’s International Statistical Classification of Diseases and Related Health Problems; ICD9 = ICD, 9th Revision, Clinical Modification (ICD-9-CM) codes; ICD10 = ICD, 10th Revision, Canada (ICD-10-CA) codes; HIV = human immunodeficiency virus; AICD = automated internal cardioverter-defibrillator, CVD = cardiovascular disease. (DOCX) [file pone.0279710.s003.docx]

**Item S3.** **ICD diagnostic codes used to define baseline comorbidities**

| **Condition** | **Codes** |
| --- | --- |
| **Myocardial infarction** | ICD9: 410, 412; ICD10: I21, I22, I252 |
| **Congestive heart failure** | ICD9: 39891, 402, 404, 425, 428; ICD10: I43, I50, I099, I110, I130, I132, I255, I420, I425, I426, I427, I428, I429, P290 |
| **Peripheral vascular disease** | ICD9: 0930, 437, 440, 441, 443, 4471, 5571, 5579, V434; ICD10: I70, I71, I731, I738, I739, I771, I790, I792, K551, K558, K559, Z958, Z959 |
| **Cerebrovascular disease** | ICD9: 36234, 430-438; ICD10: G45, G46, I60-I69, H340 |
| **Dementia** | ICD9: 290, 2941, 3312; ICD10: F00-F03, G30, F051, G311 |
| **Chronic obstructive pulmonary disease** | ICD9: 4168, 4169, 490-496, 500-505, 5064, 5081, 5088; ICD10: J40-J47, J60-J67, I278, I279, J684, J701, J703 |
| **Rheumatic disease** | ICD9: 4465, 7100-7104, 7140-7142, 7148, 725; ICD10: M05, M32-M34, M06, M315, M351, M353, M360 |
| **Peptic ulcer disease** | ICD9: 531-534; ICD10: K25-K28 |
| **Mild liver disease** | ICD9: 07022, 07023, 07032, 07033, 07044, 07054, 0706, 0709, 570, 571, 5733, 5734, 5738, 5739, V427; ICD10: B18, K73, K74, K700-K703, K709, K717, K713-K715, K760, K762- K764, K768, K769, Z944 |
| **Diabetes without complications** | ICD9: 2500-2503, 2508, 2509; ICD10: E100, E101, E106, E108, E109, E110, E111, E116, E118, E119, E120, E121, E126, E128, E129, E130, E131, E136, E138, E139, E140, E141, E146, E148, E149 |
| **Diabetes with complications** | ICD9: 2504-2507; ICD10: E102- E105, E107, E112-E115, E117, E122-E125, E127, E132-E135, E137, E142-E145, E147 |
| **Paraplegia and hemiplegia** | ICD9: 3341, 342, 343, 3440-3446, 3449; ICD10: G81, G82, G041, G114, G801, G802, G830-G834, G839 |
| **Renal disease** | ICD9: 403, 404, 582, 5830, 5831, 5832, 5834, 5836, 5837, 585, 586, 5880, V420, V451, V56; ICD10: N18, N19, N052-N057, N250, I120, I131, N032-N037, Z490-Z492, Z940, Z992 |
| **Cancer** | ICD9: 140-165, 170-172, 174-176, 179-195, 200- 208, 2386; ICD10: C00-C26, C30-C34, C37-C41, C43, C45-C58, C60-C76, C81-C85, C88, C90-C97 |
| **Moderate or severe liver disease** | ICD9: 4560-4562, 5722-5724, 5728; ICD10: K704, K711, K721, K729, K765-K767, I850, I859, I864, I982 |
| **Metastatic carcinoma** | ICD9: 196-199; ICD10: C77-C80 |
| **HIV** | ICD9: 042; ICD10: B20-B24 |
| **Syncope** | ICD9: 7802; ICD10: R55 |
| **Atrial fibrillation and flutter** | ICD9: 427; ICD10: I48 |
| **Other arrhythmias** | ICD9: 426, 427, 74686, 7850; ICD10: I44-I47, I49, R00 |
| **Presence of AICD** | ICD9: 99604, V4502, V5332; ICD10: Z9501, Z9502, Z4501, Z4502 |
| **Seizure disorders** | ICD9: 345, 78033; ICD10: G40, R5680 |
| **Obstructive sleep apnea and other sleep disorders** | ICD9: 307, 327, 3270, 32711, 32712, 3272, 32720, 32721, 32723, 32724, 32726, 32727, 32729, 3273, 32730, 32731, 32732, 32733, 32734, 32735, 32736, 32737, 32739, 32742, 32743, 3275, 32752, 32753, 32759, 3278, 7805, 78050, 78051, 78053, 78055-78059, V694; ICD10: F51, G47 |

**Item S3. ICD diagnostic codes used to define baseline comorbidities (continued)**

| **Condition** | **Codes** |
| --- | --- |
| **Traumatic brain injury** | ICD9: 310, 80009, 80019, 80029, 80039, 80049, 80059, 80069, 80079, 80089, 80099, 80109, 80119, 80129, 80139, 80149, 80159, 80169, 80179, 80189, 80199, 80309, 80319, 80329, 80339, 80349, 80359, 80369, 80379, 80389, 80399, 80409, 80419, 80429, 80439, 80449, 80459, 80469, 80479, 80489, 80499, 850, 8500, 85011, 85012, 8502-8505, 8509, 85109, 85119, 85129, 85139, 85149, 85159, 85169, 85179, 85189, 85199, 85209, 85219, 85229, 85239, 85249, 85259, 85309, 85319, 85409, 85419, V1552, V8001; ICD10: F072, S06 |
| **Psychiatric disorders** | ICD9: 295-301, 306-319; ICD10: F04-F09, F2-F9 |
| **Alcohol misuse** | ICD9: 291, 303, 305, 3575, 425, 5353, 53530, 53531, 571, 76071, 7903, 9773, 980, E860, V113; ICD10: F10, G312, G721, I426, K292, K70, K852, O354, R780, T51, X45, X65, Y15, Y90, Y91, Z502, Z714, Z721, Z8640 |
| **Other substance misuse** | ICD9: 292, 304, 305, 76073, 76075, 9650, 96501, 96502, 96509, 967, 9670, 9671, 9676, 9678, 9679, 9696, 96972, 9701, 97081, E8500, E8501, E8502, E851, E852, E8520, E8525, E8528, E8529, E8541, E9350, E9351, E9352, E937, E9370, E9371, E9376, E9378, E9379, E9396, E9401, E9501, E9502, E9801, E9802; ICD10: F11-F19 |
| **Chronic ischemic heart disease** | ICD9: 414; ICD10: I25 |
| **Hypertension** | ICD9: 36211, 401-405, 6420, 64200-64204, 6421, 64210-64214, 6422, 64220-64224, 6427, 64270-64274; ICD10: I10-I15, O10, O11 |
| **Pacemaker** | ICD9: V4501, V5331; ICD10: Z4500, Z9500, Z9502, Z4502 |
| **Unstable angina** | ICD10: I200 |
| **Cardiovascular disease (CVD)** | Codes from myocardial infarction, chronic ischemic heart disease, peripheral vascular disease, congestive heart failure, atrial fibrillation and flutter, other cardiac arrhythmia, cerebrovascular disease |
