# Supplementary material for: Syncope and subsequent traffic crash: A responsibility analysis
Source: PLoS One. 2023 Jan 19;18(1):e0279710. doi: 10.1371/journal.pone.0279710 (PMC9851499; doi:10.1371/journal.pone.0279710)
Supplement: S4 File — * indicates the referent category used in regression analyses. We used a logistic regression model to evaluate the association between crash responsibility and prior ED visit for syncope. The equation took the form logit[Pr(Y=1|X=x)]=β0+βsxs+∑j=1kβjxj where Y = Driver responsibility for crash (1 = responsible, 0 = non-responsible) β0 = Intercept βs = Regression coefficient that represents the change in the log odds of crash responsibility for drivers with versus without an emergency department visit for ’syncope and collapse’ in the 3 months prior to the index crash xs = Indicator variable for whether the driver had an emergency department visit for ’syncope and collapse’ in the 3 months prior to the index crash (1 = present, 0 = absent) βj = Regression coefficient representing the change in the log odds of crash responsibility for a 1-unit increase in the jth potential confounder xj = Variable representing the value of the jth potential confounder k = Number of potential confounders. (DOCX) [file pone.0279710.s004.docx]

**Item S4. Variable definitions**

| **Variables** | **Definition** |
| --- | --- |
| **Outcome** |  |
| Crash responsibility | Crash responsibility was determined using objective police data and a validated responsibility scoring tool. Responsibility scores were categorized as 'responsible' (score ≤13), 'non-responsible' (score ≥16), or indeterminate (score 14 - 15) in the main analysis. |
| **Exposures** |  |
| Syncope | An emergency department visit with a discharge diagnosis of 'syncope and collapse' that occurred in the exposure lookback interval. The main analysis used a 3-monoth exposure lookback interval. |
| Syncope 'definite or likely' | Among individuals with syncope as defined above, those in whom syncope was deemed by trained chart abstractors to be 'definite' or 'very likely' based on comprehensive review of medical records from the index emergency department visit. |
| **Potential confounders** |  |
| **Demographic** |  |
| Age group | Driver age at index crash date (16-35 years; 36-55 years*; ≥56 years) |
| Sex | Driver sex (male; female*) |
| Residential neighbourhood  household income  quintile | Driver residential neighbourhood income quintile generated by PopDataBC using census data and driver residential postal code (1 = lowest*, 5 = highest; ordinal) |
| **Driving history** |  |
| License type | Type of driver license held at the time of index crash (full vs novice or learner*) |
| Years with automobile  insurance in past 5y | Number of years with auto insurance in the 5 years before index crash (0 years*, >0 to ≤ 2.5 years, 2.5 to 5 years) |
| ≥1 contravention in the  past 5y | Traffic violations include speeding, distracted driving, or impaired driving in the 5 years before index crash (yes/no). |
| ≥1 crash in the past 5y | Police-reported crash in the 5 years before index crash (yes/no). |
| **Crash characteristics** |  |
| Crash season | Season of index crash: Winter (Dec- Feb); Spring (Mar- May); Summer (Jun-Aug); Fall (Sep-Nov)* |
| Substance impairment at  time of crash | Breath or blood test positive for alcohol, or alcohol or drug impairment suspected by police officer (yes/no) |
| Annual percent responsible  by crash year | Of all police-reported crashes in BC, the proportion of crashes deemed ‘responsible’ in the index crash year (continuous variable)^40^ |
| **Health** |  |
| CCI in the past year | Dichotomized variable for Charlson Comorbidity Index (CCI) ≥2 (yes/no); Comorbidities were deemed to be present if the associated diagnostic codes were found in ≥1 hospitalization or ≥2 clinic visits in the year before index crash |
| Hospitalization in the past  year | Dichotomized variable for ≥1 hospitalizations in the year before index crash (yes/no). |
| Physician visits in the past  year | Number of physician/clinic visits in the year before index crash; 0 visits*, ≤ 1 per month (1-12 visits); ≤ 1 per week (13-52 visits); > 1 per week (>52 visits) |
| History of cardiovascular  disease in past year | ≥1 hospitalization or ≥2 clinic visits for cardiovascular disease in the year before index crash (yes/no) |
| History of diabetes in past  year | ≥1 hospitalization or ≥2 clinic visits for diabetes in the year before index crash (yes/no) |
| History of alcohol misuse  in past year | ≥1 hospitalization or ≥2 clinic visits for alcohol use disorder in the year before index crash (yes/no) |
| History of other substance  misuse in past year | ≥1 hospitalization or ≥2 clinic visits for other substance use disorder in the year before index crash (yes/no) |
| Presence of AICD or  pacemaker in past year | Presence of diagnostic codes for automated internal cardioverter-defibrillator (AICD) or pacemaker in hospital or clinic visit records in the year before index crash (yes/no) |
| Number of distinct  medications prescribed  within 60 days prior to  crash | Number of distinct prescription medications likely consumed in the 60 days prior to index crash excluding those prescribed on the index crash date (0*, 1, ≥2 distinct medications). Overlap with 60-day lookback determined using dispensation date and days supplied. |
| Benzodiazepines prescribed  within 60 days prior to  crash | Prescription for benzodiazepines dispensed in the 60 days prior to index crash, excluding index crash date (yes/no). |
| Opioids prescribed within  60 days prior to crash | Prescription for opioids dispensed in the 60 days prior to index crash, excluding index crash date (yes/no). |
