# Supplementary figures and images for: Syncope and subsequent traffic crash: A responsibility analysis
Source: PLoS One. 2023 Jan 19;18(1):e0279710. doi: 10.1371/journal.pone.0279710 (PMC9851499; doi:10.1371/journal.pone.0279710)

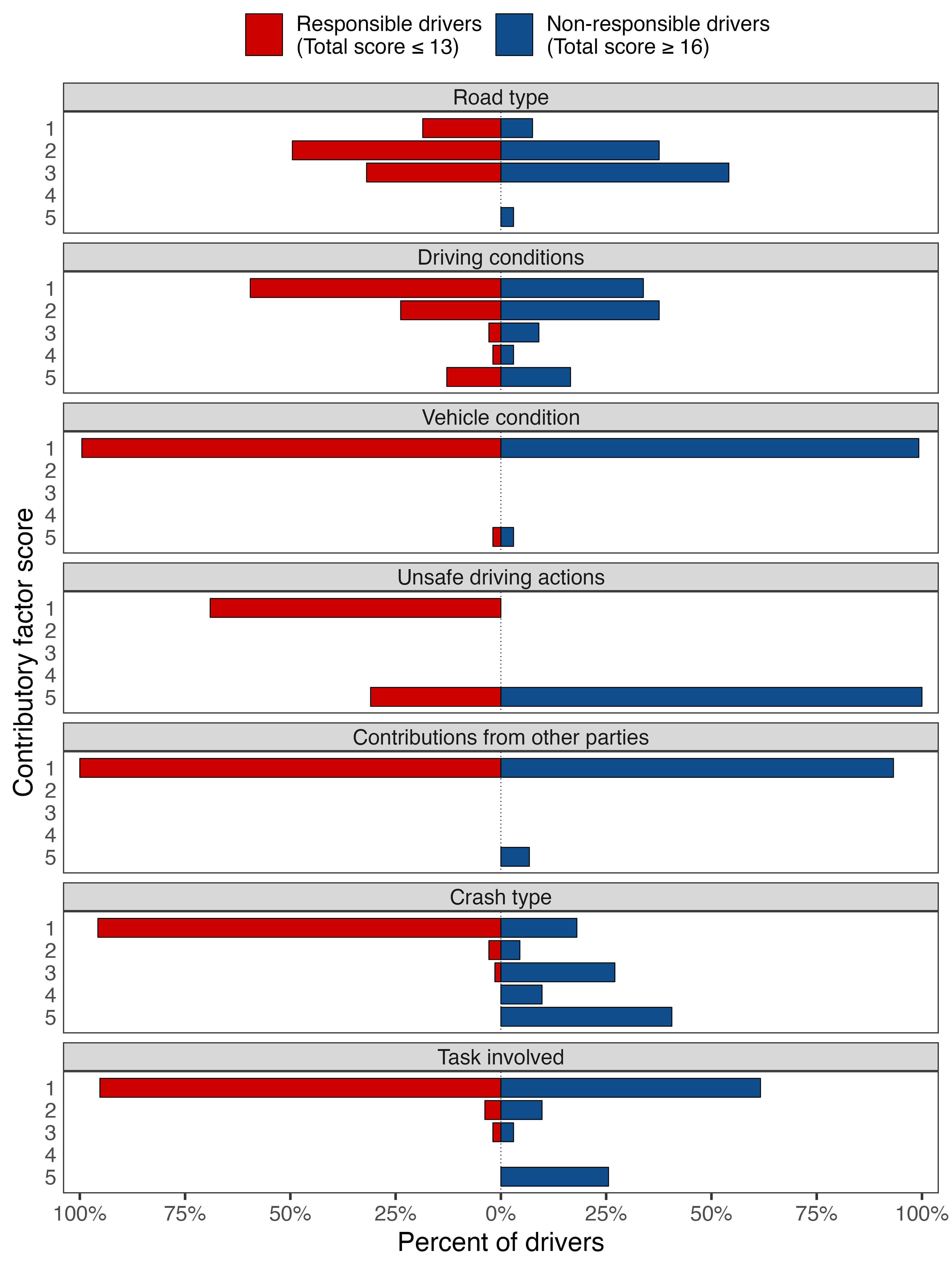

Supplement: S5 File — Mirrored bar chart comparing scores between responsible and non-responsible drivers for each of the seven components of the responsibility score. (TIF) [file pone.0279710.s005.tif]

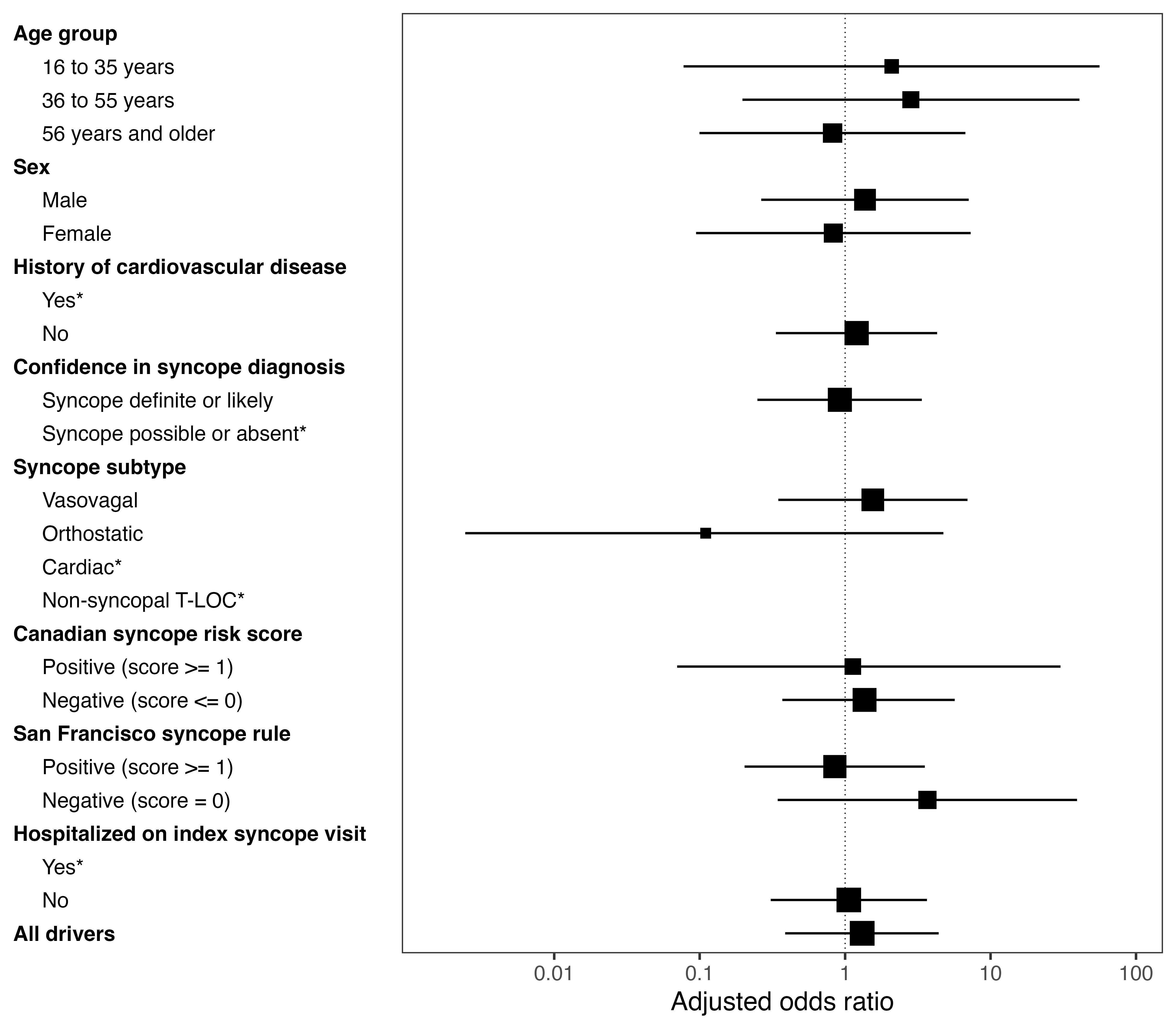

Supplement: S7 File — X-axis depicts the adjusted odds ratio for the association between syncope and crash responsibility; y-axis, the subgroup; square points, the adjusted odds ratio point estimate (with size reflecting the inverse of the standard error); horizontal lines, the 95% confidence interval, with arrow heads indicating the confidence interval endpoint is beyond the limit of the x-axis. Syncope was not associated with crash responsibility in any subgroup. *Indicates that all drivers in these strata were deemed responsible for their crash, making it impossible to calculate an odds ratio. (TIF) [file pone.0279710.s007.tif]
