# Supplementary material for: Syncope and subsequent traffic crash: A responsibility analysis
Source: PLoS One. 2023 Jan 19;18(1):e0279710. doi: 10.1371/journal.pone.0279710 (PMC9851499; doi:10.1371/journal.pone.0279710)
Supplement: S6 File — Table depicting crash characteristics for responsible and non-responsible drivers. These crash characteristics are reported by police on form MV6020 and aggregated within BC’s Traffic Accident System dataset. An example of the data collection form can be found online (Traffic Crash Reports & Overlay Forms [Internet]. North Platte (NE): Accreditation Commission for Traffic Accident Reconstruction; 2022. Accessed 14 Nov 2022 at https://actar.org/resources/reports). We had detailed police-reported crash data for all syncope cohort members but lacked crash data for other drivers involved in the crash. As a result, except where the index driver’s data directly suggested contribution from others (e.g., pedestrian error, previous traffic crash), the ’contributions from other parties’ factor could not account for other drivers’ actions. This may have biased responsibility scores downward and effect estimates toward the null. However, the proportion of crash-involved drivers deemed responsible was similar to that within the largest prior responsibility study (44.2% versus 46%, respectively), suggesting missing data on ’contributions from other parties’ had a limited effect on our results (Brubacher JR, Chan H, Erdelyi S, Zed PJ, Staples JA, Etminan M. Medications and risk of motor vehicle collision responsibility in British Columbia, Canada: a population-based case-control study. Lancet Public Health. 2021 Jun;6(6):e374-e385.). Not all displayed data are components of the responsibility score tool. As expected, responsible drivers were more likely to be disobeying road laws and were more likely to be involved in crashes that occurred on dry roads, during optimal weather, in full daylight, and involving only a single vehicle (these are components of the responsibility score). Reassuringly, established risk factors for crash that are not part of the responsibility score are also more common among responsible drivers. For example, human contributory factors, which include alcoh [file pone.0279710.s006.docx]

**Item S6. Crash characteristics among responsible and non-responsible drivers**

| **Characteristic** | **Responsible  n = 210 (100%)** | **Non‐responsible  n = 133 (100%)** | **Indeterminate  n = 132 (100%)** | **p-value** (responsible  vs. non-responsible) |
| --- | --- | --- | --- | --- |
| **Road type** |  |  |  |  |
| Traffic flow |  |  |  |  |
| One-way | 32 (15.2%) | 9 (6.8%) | 12 (9.1%) | 0.02 |
| Two-way | 171 (81.4%) | 123 (92.5%) | 116 (87.9%) |  |
| Other/unknown | 7 (3.3%) | < 5 | < 5 |  |
| Road class |  |  |  |  |
| Single lane | 6 (2.9%) | 6 (4.5%) | 7 (5.3%) | 0.02 |
| Single lane, ramp |  |  |  |  |
| Multi-lane | 159 (75.7%) | 106 (79.7%) | 106 (80.3%) | 0.02 |
| Multi-lane, ramp | 0 | 0 | < 5 |  |
| Not applicable | 30 (14.3%) | 6 (4.5%) | < 5 |  |
| Other/unknown | 15 (7.1%) | 15 (11.3%) | 14 (10.6%) |  |
| Accident location |  |  |  |  |
| At intersection | 73 (34.8%) | 75 (56.4%) | 56 (42.4%) | <0.0001 |
| Between intersection | 74 (35.2%) | 45 (33.8%) | 48 (36.4%) |  |
| Parking lot | 22 (10.5%) | < 5 | < 5 |  |
| Other/unknown | 41 (19.5%) | 11 (8.3%) | 25 (18.9%) |  |
| Roadside hazard or road design  listed as contributory factor | 0 | < 5 | 0 | 0.82 |
| **Driving conditions** |  |  |  |  |
| Road condition |  |  |  |  |
| Dry | 137 (65.2%) | 52 (39.1%) | 97 (73.5%) | <0.0001 |
| Wet | 62 (29.5%) | 70 (52.6%) | 28 (21.2%) |  |
| Snow/slush/ice/mud | 9 (4.3%) | 10 (7.5%) | < 5 |  |
| Other/unknown | < 5 | < 5 | < 5 |  |
| Road surface |  |  |  |  |
| Asphalt/concrete | 208 (99.0%) | 133 (100.0%) | 130 (98.5%) | 0.69 |
| Stone/gravel/earth/wood | < 5 | 0 | < 5 |  |
| Other/unknown | 0 | 0 | < 5 |  |
| Weather |  |  |  |  |
| Clear/cloudy | 161 (76.7%) | 76 (57.1%) | 110 (83.3%) | 0.003 |
| Rain/strong wind | 38 (18.1%) | 48 (36.1%) | 16 (12.1%) |  |
| Fog/smoke/smog | < 5 | < 5 | 0 |  |
| Snow/sleet/hail | 5 (2.4%) | 5 (3.8%) | < 5 |  |
| Other/unknown | < 5 | < 5 | < 5 |  |
| Lighting |  |  |  |  |
| Daylight | 145 (69.0%) | 84 (63.2%) | 99 (75.0%) | 0.49 |
| Dusk/dawn | 18 (8.6%) | 8 (6.0%) | 5 (3.8%) |  |
| Dark with full illumination | 20 (9.5%) | 17 (12.8%) | 17 (12.9%) |  |
| Dark with no/some  illumination | 26 (12.4%) | 23 (17.3%) | 10 (7.6%) |  |
| Other/unknown | < 5 | < 5 | < 5 |  |
| Weather or visibility listed as  contributory factor | 27 (12.9%) | 22 (16.5%) | < 5 | 0.43 |

**Item S6. Crash characteristics among responsible and non-responsible drivers (continued)**

| **Characteristic** | **Responsible  n = 210 (100%)** | **Non‐responsible  n = 133 (100%)** | **Indeterminate  n = 132 (100%)** | **p-value** (responsible  vs. non-responsible) |
| --- | --- | --- | --- | --- |
| **Vehicle condition** |  |  |  |  |
| Vehicle condition not listed as contributory factor | 209 (99.5%) | 132 (99.2%) | 131 (99.2%) | 1.00 |
| **Unsafe driving actions** |  |  |  |  |
| Index driver driving safely and  obeying road laws | 65 (31.0%) | 133 (100.0%) | 132 (100.0%) | <0.0001 |
| Index driver not driving safely  or disobeying road laws | 145 (69.0%) | 0 | 0 |  |
| **Contributions from other parties** |  |  |  |  |
| Yes | 0 | 9 (6.8%) | 0 | 0.001 |
| No / index driver driving  unsafely | 210 (100.0%) | 124 (93.2%) | 132 (100.0%) |  |
| **Crash type** |  |  |  |  |
| Number of vehicles involved |  |  |  |  |
| Single vehicle | 55 (26.2%) | 23 (17.3%) | 12 (9.1%) | 0.02 |
| Two vehicles | 116 (55.2%) | 70 (52.6%) | 93 (70.5%) |  |
| Three or more vehicles | 39 (18.6%) | 40 (30.1%) | 27 (20.5%) |  |
| Diagram description |  |  |  |  |
| Intersection - right angle | 27 (12.9%) | 34 (25.6%) | 19 (14.4%) | 0.004 |
| Head on | 22 (10.5%) | 8 (6.0%) | 18 (13.6%) |  |
| Rear end | 53 (25.2%) | 49 (36.8%) | 14 (10.6%) |  |
| Backing | 6 (2.9%) | < 5 | < 5 |  |
| Turn | 23 (11.0%) | 11 (8.3%) | 30 (22.7%) |  |
| Overtaking | 8 (3.8%) | < 5 | 13 (9.8%) |  |
| Off road | 23 (11.0%) | 8 (6.0%) | 7 (5.3%) |  |
| Other/unknown | 48 (22.9%) | 18 (13.5%) | 29 (22.0%) |  |
| Damage location |  |  |  |  |
| Front | 117 (55.7%) | 37 (27.8%) | 48 (36.4%) | <0.0001 |
| Front/rear | 10 (4.8%) | 5 (3.8%) | 5 (3.8%) |  |
| Front/side | 10 (4.8%) | 10 (7.5%) | 17 (12.9%) |  |
| Rear | 10 (4.8%) | 37 (27.8%) | 17 (12.9%) |  |
| Rear/side | < 5 | < 5 | < 5 |  |
| Side | 5 (2.4%) | 0 | < 5 |  |
| Tires/undercarriage/  windshield/roof | 13 (6.2%) | 10 (7.5%) | 10 (7.6%) |  |
| Whole vehicle | 8 (3.8%) | 7 (5.3%) | 8 (6.1%) |  |
| None | 10 (4.8%) | 8 (6.0%) | 7 (5.3%) |  |
| Other/unknown | 117 (55.7%) | 37 (27.8%) | 48 (36.4%) | <0.0001 |
| Pre-collision action |  |  |  |  |
| Straight | 124 (59.0%) | 81 (60.9%) | 81 (61.4%) | <0.0001 |
| Backing | 7 (3.3%) | < 5 | < 5 |  |
| Turning | 49 (23.3%) | 12 (9.0%) | 36 (27.3%) |  |
| Changing lanes/merging | < 5 | < 5 | < 5 |  |
| Loss of control | 6 (2.9%) | 0 | < 5 |  |
| Stopped/parked | 0 | 26 (19.5%) | < 5 |  |
| Other/unknown | 20 (9.5%) | 11 (8.3%) | 7 (5.3%) |  |

**Item S6. Crash characteristics among responsible and non-responsible drivers (continued)**

| **Characteristic** | **Responsible  n = 210 (100%)** | **Non‐responsible  n = 133 (100%)** | **Indeterminate  n = 132 (100%)** | **p-value** (responsible  vs. non-responsible) |
| --- | --- | --- | --- | --- |
| **Task involved** |  |  |  |  |
| Avoidance manoeuvre listed as  contributory factor | < 5 | 8 (6.0%) | < 5 | 0.005 |
| Pre-collision action |  |  |  |  |
| Straight | 124 (59.0%) | 81 (60.9%) | 81 (61.4%) | <0.0001 |
| Backing | 7 (3.3%) | < 5 | < 5 |  |
| Turning | 49 (23.3%) | 12 (9.0%) | 36 (27.3%) |  |
| Changing lanes/merging | < 5 | < 5 | < 5 |  |
| Loss of control | 6 (2.9%) | 0 | < 5 |  |
| Stopped/parked | 0 | 26 (19.5%) | < 5 |  |
| Other/unknown | 20 (9.5%) | 11 (8.3%) | 7 (5.3%) |  |
| **Other crash characteristics** |  |  |  |  |
| Human condition listed as  contributory factor | 114 (54.3%) | 22 (16.5%) | 44 (33.3%) | <0.0001 |
| Alcohol | 11 (5.2%) | < 5 | < 5 |  |
| Medications | < 5 | 0 | 0 |  |
| Drugs | < 5 | 0 | < 5 |  |
| Illness/fatigue | 22 (10.5%) | < 5 | 7 (5.3%) |  |
| Distracted/inattentive | 78 (37.1%) | 16 (12.0%) | 31 (23.5%) |  |
| Pre-Existing Physical Disability | < 5 | < 5 | < 5 |  |
| Breath alcohol positive |  |  |  |  |
| Yes | < 5 | 0 | < 5 | 1.00 |
| No/not tested | 209 (99.5%) | 133 (100.0%) | 131 (99.2%) |  |
| Accident severity |  |  |  |  |
| Casualty (fatal or injury) | 104 (49.5%) | 63 (47.4%) | 63 (47.7%) | 0.78 |
| Property damage | 106 (50.5%) | 70 (52.6%) | 69 (52.3%) |  |
| Road location |  |  |  |  |
| Rural road | < 5 | 0 | < 5 | 0.43 |
| Provincial highway | 49 (23.3%) | 25 (18.8%) | 15 (11.4%) |  |
| City street | 160 (76.2%) | 108 (81.2%) | 116 (87.9%) |  |
| Speed zone |  |  |  |  |
| < 50 km/h | 14 (6.7%) | < 5 | < 5 | 0.18 |
| 50 km/h | 117 (55.7%) | 89 (66.9%) | 101 (76.5%) |  |
| 60 - 70 km/h | 24 (11.4%) | 12 (9.0%) | 13 (9.8%) |  |
| >= 80 km/h | 29 (13.8%) | 17 (12.8%) | 5 (3.8%) |  |
| Other/unknown | 26 (12.4%) | 12 (9.0%) | 10 (7.6%) |  |
| Road character |  |  |  |  |
| Straight - flat | 128 (61.0%) | 81 (60.9%) | 75 (56.8%) | 0.20 |
| Straight - graded | 46 (21.9%) | 26 (19.5%) | 34 (25.8%) |  |
| Curved - flat |  |  |  |  |
| Curved - graded |  |  |  |  |
| Other/unknown | 9 (4.3%) | 14 (10.5%) | 7 (5.3%) | 0.20 |

**Item S6. Crash characteristics among responsible and non-responsible drivers (continued)**

| **Characteristic** | **Responsible  n = 210 (100%)** | **Non‐responsible  n = 133 (100%)** | **Indeterminate  n = 132 (100%)** | **p-value** (responsible  vs. non-responsible) |
| --- | --- | --- | --- | --- |
| **Other crash characteristics (cont'd)** |  |  |  |  |
| Vehicle damage severity |  |  |  |  |
| Light | 47 (22.4%) | 43 (32.3%) | 35 (26.5%) | 0.01 |
| Moderate | 58 (27.6%) | 37 (27.8%) | 29 (22.0%) |  |
| Severe | 67 (31.9%) | 24 (18.0%) | 39 (29.5%) |  |
| Demolished | 19 (9.0%) | 7 (5.3%) | 8 (6.1%) |  |
| None | 8 (3.8%) | 7 (5.3%) | 8 (6.1%) |  |
| Other/unknown | 11 (5.2%) | 15 (11.3%) | 13 (9.8%) |  |
| Crash year |  |  |  |  |
| 2010 | 44 (21.0%) | 22 (16.5%) | 20 (15.2%) | 0.33 |
| 2011 | 32 (15.2%) | 33 (24.8%) | 26 (19.7%) |  |
| 2012 | 49 (23.3%) | 31 (23.3%) | 27 (20.5%) |  |
| 2013 | 29 (13.8%) | 16 (12.0%) | 30 (22.7%) |  |
| 2014 | 25 (11.9%) | 16 (12.0%) | 15 (11.4%) |  |
| 2015 | 27 (12.9%) | 11 (8.3%) | 12 (9.1%) |  |
| 2016 | < 5 | < 5 | < 5 |  |
| Crash season |  |  |  |  |
| Winter (Dec-Feb) | 53 (25.2%) | 48 (36.1%) | 38 (28.8%) | 0.09 |
| Spring (Mar-May) | 52 (24.8%) | 31 (23.3%) | 27 (20.5%) |  |
| Summer (Jun-Aug) | 54 (25.7%) | 22 (16.5%) | 39 (29.5%) |  |
| Fall (Sep-Nov) | 51 (24.3%) | 32 (24.1%) | 28 (21.2%) |  |
| Day of week |  |  |  |  |
| Weekday (Mon-Thur) | 119 (56.7%) | 75 (56.4%) | 91 (68.9%) | 1.00 |
| Weekend (Fri-Sun) | 91 (43.3%) | 58 (43.6%) | 41 (31.1%) |  |
| Time of day |  |  |  |  |
| Morning (06:01-12:00) | 47 (22.4%) | 25 (18.8%) | 34 (25.8%) | 0.70 |
| Afternoon (12:01-18:00) | 107 (51.0%) | 70 (52.6%) | 63 (47.7%) |  |
| Evening (18:01-21:00) | 32 (15.2%) | 17 (12.8%) | 14 (10.6%) |  |
| Night (21:01-06:00) | 21 (10.0%) | 19 (14.3%) | 15 (11.4%) |  |
| Other/unknown | < 5 | < 5 | 6 (4.5%) |  |
