# Supplementary material for: Syncope and subsequent traffic crash: A responsibility analysis
Source: PLoS One. 2023 Jan 19;18(1):e0279710. doi: 10.1371/journal.pone.0279710 (PMC9851499; doi:10.1371/journal.pone.0279710)
Supplement: S8 File — *Could not be estimated because all exposed drivers were responsible. ** Too few crashes to estimate parameters in adjusted model. (DOCX) [file pone.0279710.s008.docx]

**Item S8. Sensitivity analysis of exposure lookback period for selected subgroups**

| **Subgroup** | **% With an ED visit for syncope among responsible drivers** | **% With an ED visit for syncope among non-responsible drivers** | **Unadjusted** | | **Adjusted** | |
| --- | --- | --- | --- | --- | --- | --- |
|  |  |  | **OR**  **(95% CI)** | **p** | **OR**  **(95% CI)** | **p** |
| **History of cardiovascular disease** |  |  |  |  |  |  |
| ED visit within 1 month | 0.0% (0/25) | 0.0% (0/12) | * | * | * | * |
| ED visit within 3 months | 8.0% (<5) | 0.0% (0/12) | * | * | * | * |
| ED visit within 6 months | 12.0% (<5) | 8.3% (<5) | 1.50  (0.17, 32.3) | 0.74 | ** | ** |
| ED visit within 9 months | 20.0% (5/25) | 16.7% (<5) | 1.25  (0.22, 9.81) | 0.81 | ** | ** |
| ED visit within 12 months | 24.0% (6/25) | 25.0% (<5) | 0.95  (0.20, 5.29) | 0.95 | ** | ** |
| **Cardiac syncope** |  |  |  |  |  |  |
| ED visit within 1 month |  |  |  |  |  |  |
| ED visit within 3 months | 1.0% (<5) | 0% (0/128) | * | * | * | * |
| ED visit within 6 months | 1.1% (<5) | 0% (0/115) | * | * | * | * |
| ED visit within 9 months | 1.1% (<5) | 0.9% (<5) | 1.22  (0.12, 26.39) | 0.87 | 1.16  (0.06, 35.92) | 0.92 |
| ED visit within 12 months | 1.2% (<5) | 1.0% (<5) | 1.22  (0.12, 26.42) | 0.87 | 1.04  (0.06, 31.81) | 0.98 |

**Item S8. Sensitivity analysis of exposure lookback period for selected subgroups (continued)**

| **Subgroup** | **% With an ED visit for syncope among responsible drivers** | **% With an ED visit for syncope among non-responsible drivers** | **Unadjusted** | | **Adjusted** | |
| --- | --- | --- | --- | --- | --- | --- |
|  |  |  | **OR**  **(95% CI)** | **p** | **OR**  **(95% CI)** | **p** |
| **Hospitalized on index syncope visit** |  |  |  |  |  |  |
| ED visit within 1 month | 0.5% (<5) | 0.0% (0/132) | * | * | * | * |
| ED visit within 3 months | 0.5% (<5) | 0.0% (0/128) | * | * | * | * |
| ED visit within 6 months | 1.1% (<5) | 0.9% (<5) | 1.25  (0.12, 27.1) | 0.86 | 3.36  (0.25, 85.4) | 0.37 |
| ED visit within 9 months | 1.1% (<5) | 0.9% (<5) | 1.22  (0.12, 26.4) | 0.87 | 3.02  (0.22, 76.9) | 0.42 |
| ED visit within 12 months | 1.2% (<5) | 1.0% (<5) | 1.22  (0.12, 26.4) | 0.87 | 2.75  (0.20, 70.9) | 0.46 |
