# Supplementary material for: Syncope and subsequent traffic crash: A responsibility analysis
Source: PLoS One. 2023 Jan 19;18(1):e0279710. doi: 10.1371/journal.pone.0279710 (PMC9851499; doi:10.1371/journal.pone.0279710)
Supplement: S9 File — Main effect is bolded and highlighted in blue. OR = odds ratio, CI = confidence interval, ref = reference category, ED = Emergency Department, BC = British Columbia, AICD = Automated internal cardioverter-defibrillator. (DOCX) [file pone.0279710.s009.docx]

**Item S9. Regression coefficients for the main analysis of syncope and crash responsibility**

| **Variable** | **OR (95% CI)** | **p value** |
| --- | --- | --- |
| (Intercept) | 1.33 (0.22, 8.26) | 0.76 |
| **Index ED visit for syncope** | **1.31 (0.40, 4.74)** | **0.67** |
| Age group (ref = 36-55 years) |  |  |
| 16-35 years | 0.70 (0.34, 1.43) | 0.32 |
| ≥ 56 years | 0.89 (0.45, 1.72) | 0.72 |
| Male | 1.49 (0.89, 2.50) | 0.13 |
| Neighborhood income quintile (ref = first) |  |  |
| Second | 1.16 (0.54, 2.49) | 0.70 |
| Third | 1.34 (0.60, 3.00) | 0.48 |
| Fourth | 1.34 (0.61, 2.96) | 0.47 |
| Fifth | 1.63 (0.77, 3.46) | 0.20 |
| Annual percent responsible by crash year in BC (%) | 1.06 (0.68, 1.64) | 0.81 |
| Crash season (ref = Fall) |  |  |
| Spring | 0.99 (0.50, 1.97) | 0.98 |
| Summer | 1.55 (0.76, 3.20) | 0.24 |
| Winter | 0.67 (0.35, 1.28) | 0.23 |
| Substance impairment suspected or breath test positive for alcohol at time of crash | 4.22 (0.91, 33.60) | 0.10 |
| ≥ 1 contravention in the past 5 years | 1.90 (1.12, 3.24) | 0.02 |
| ≥ 1 crash in the past 5 years | 0.72 (0.43, 1.22) | 0.23 |
| Full license (ref = novice or learner) | 0.46 (0.19, 1.05) | 0.07 |
| Number of years with auto insurance in past 5 years (ref = 0 years) |  |  |
| ≤ 2.5 years | 0.48 (0.20, 1.11) | 0.09 |
| 2.5-5 years | 0.86 (0.39, 1.83) | 0.70 |
| CCI ≥ 2 | 0.72 (0.24, 2.11) | 0.55 |
| ≥ 1 hospitalizations in the past year | 0.97 (0.40, 2.36) | 0.95 |
| Number of physician visits in the past year (ref = 0 visits) |  |  |
| ≤ 1 per month (1-12 visits) | 1.46 (0.40, 5.20) | 0.56 |
| ≤ 1 per week (13-52 visits) | 1.06 (0.27, 4.18) | 0.93 |
| > 1 per week (> 52 visits) | 0.78 (0.12, 5.45) | 0.80 |
| History of cardiovascular disease in past year | 1.11 (0.42, 3.13) | 0.84 |
| History of diabetes in past year | 0.87 (0.22, 3.41) | 0.83 |
| History of alcohol misuse in past year | 0.80 (0.10, 5.90) | 0.83 |
| History of other substance misuse in past year | 7.30 (0.68, 201.5) | 0.15 |
| Presence of AICD or pacemaker in past year | 0.59 (0.07, 5.61) | 0.62 |

**Item S9. Regression coefficients for the main analysis of syncope and crash responsibility (continued)**

| **Variable** | **OR (95% CI)** | **p value** |
| --- | --- | --- |
| Number of prescription medications prescribed within 60 days of crash (ref = None) |  |  |
| One | 1.48 (0.71, 3.13) | 0.30 |
| At least two | 2.06 (1.01, 4.27) | 0.05 |
| Benzodiazepines prescribed within 60 days of crash | 0.74 (0.25, 2.19) | 0.58 |
| Opioids prescribed within 60 days of crash | 1.23 (0.45, 3.57) | 0.69 |
