# Supplementary material for: Syncope and subsequent traffic crash: A responsibility analysis
Source: PLoS One. 2023 Jan 19;18(1):e0279710. doi: 10.1371/journal.pone.0279710 (PMC9851499; doi:10.1371/journal.pone.0279710)
Supplement: Attachment — Submitted filename: Syncope-MVC_resp - PLoS R2R v4.docx [file pone.0279710.s010.docx]

**Author Response Letter for “**Syncope and subsequent traffic crash: A responsibility analysis**” (Manuscript PONE-D-22-23799, PLoS ONE) accompanying the first revision.**

Editor and reviewer comments in bold; author responses in italics.

**EDITOR'S COMMENTS:**

**Editor: Thank you for submitting your manuscript to PLOS ONE ... we invite you to submit a revised version of the manuscript that addresses the points raised during the review process.**

*AUTHORS' RESPONSE: We thank the editor for inviting us to submit a revision. We have made the suggested changes including formatting to be consistent with PLOS ONE style templates.*

**Editor: Thank you for stating the following in the Acknowledgments Section of your manuscript:**

**"This study was supported by the Canadian Institutes of Health Research (grant number PJT-148849). JS' salary was supported by the Vancouver Coastal Health Research Institute and by a Health Professional-Investigator Award from Michael Smith Health Research"**

**We note that you have provided funding information that is not currently declared in your Funding Statement. ... let us know how you would like to update your Funding Statement. Currently, your Funding Statement reads as follows:**

**"This study was supported by the Canadian Institutes of Health Research (grant number PJT-148849; cihr-irsc.gc.ca). JS' salary was supported by the Vancouver Coastal Health Research Institute (vchri.ca) and by a Health Professional-Investigator Award from Michael Smith Health Research BC (msfhr.org). JB was supported by Michael Smith Health Research BC and the British Columbia Emergency Medicine Network (bcemergencynetwork.ca). The funders had no role in study design, data collection and analysis, decision to publish, or preparation of the manuscript."**

**Please include your amended statements within your cover letter; we will change the online submission form on your behalf.**

*AUTHORS' RESPONSE: We have removed all funding-related text from our manuscript. The Funding Statement contains all details that were previously noted in the Acknowledgements section and contains all funding information for this study.*

**Editor: In your Data Availability statement, you have not specified where the minimal data set underlying the results described in your manuscript can be found. PLOS defines a study's minimal data set as the underlying data used to reach the conclusions drawn in the manuscript and any additional data required to replicate the reported study findings in their entirety. All PLOS journals require that the minimal data set be made fully available. For more information about our data policy, please see http://journals.plos.org/plosone/s/data-availability.**

**Upon re-submitting your revised manuscript, please upload your study’s minimal underlying data set as either Supporting Information files or to a stable, public repository and include the relevant URLs, DOIs, or accession numbers within your revised cover letter. For a list of acceptable repositories, please see http://journals.plos.org/plosone/s/data-availability#loc-recommended-repositories. Any potentially identifying patient information must be fully anonymized.**

**Important: If there are ethical or legal restrictions to sharing your data publicly, please explain these restrictions in detail. Please see our guidelines for more information on what we consider unacceptable restrictions to publicly sharing data: http://journals.plos.org/plosone/s/data-availability#loc-unacceptable-data-access-restrictions. Note that it is not acceptable for the authors to be the sole named individuals responsible for ensuring data access.**

**We will update your Data Availability statement to reflect the information you provide in your cover letter.**

*AUTHORS' RESPONSE: The data sets linked for use in this study are owned by the Data Stewards listed in Appendix Item S2. We now describe data access in the manuscript as follows:*

*"The analyses presented in this paper are based on laboratory and healthcare utilization datasets which are available upon approval from the respective Data Stewards. Researchers who meet the criteria for access to confidential data can accesses the data used for this study through Population Data BC. More information on data access procedure is available at: https://www.popdata.bc.ca/data_access."*

**Reviewer #1: It is an interesting study. The authors shown us clearly the data, method and results, we can learn a lot of information from the study. Be sure to say something that is insufficient, maybe the authors should be put figures and table near its corresponding descriptions. This maybe the requirement of the Journal, but which real bring inconvenient for readers.**

*AUTHORS' RESPONSE: We thank Reviewer #1 for their appreciative comments. We happily leave decisions regarding formatting to the Editor and to the PLoS ONE team.*

**Reviewer #2: This paper focused on the syncope and subsequent traffic crash, based on the collision investigation report to analyze. The topic is interesting, while there are some issues should be modified. Some suggestions:**

**1.Authors should provide the samples for the collision investigation report with detailed information.**

*AUTHORS' RESPONSE: We thank Reviewer#2 for finding our study interesting.*

*We have now indicated in our appendix (p11) that the detailed data we present in Item S6 is all derived from police crash reports. We now state the number of the police crash report form and provide a reference that allows readers to obtain a version of the reporting form:*

*"These crash characteristics are reported by police on form MV6020 and aggregated within BC's Traffic Accident System dataset. An example of the data collection form can be found online. [Traffic Crash Reports & Overlay Forms [Internet]. North Platte (NE): Accreditation Commission for Traffic Accident Reconstruction; 2022. Accessed 14 Nov 2022 at https://actar.org/resources/reports]"*

**2. Authors mentioned “used logistic regression to evaluate the association between crash responsibility and prior ED visit for syncope.” Here should provide the formula and results of the logistic regression model.**

*AUTHORS' RESPONSE: We now provide the logistic regression formula for interested readers in Supplemental Appendix (Item S4, p6). We now also provide the results of the main logistic regression model in the Supplemental Appendix (Item S9, p16).*

**3. Authors find that “no significant association between an emergency department visit for syncope and driver responsibility for a subsequent motor vehicle crash”, there are many influencing factors of traffic crashes, how to screen the influence of drivers, road and environmental factors needs to be further explored.**

*AUTHORS' RESPONSE: We agree that many factors contribute to crash responsibility. Our revised submission now quantifies the influence of important confounders by presenting the regression coefficients for readers to review (Supplemental Appendix, Item S9, p16). This includes many driver characteristics known to influence crash risk. Road and environmental factors that contribute to the likelihood of crash are also presented in detail in our revised submission (Supplemental Appendix, Item S6, p8). We highlight study limitations including incomplete data on additional potential confounders in our Discussion (p18).*
